# Cancer Cure Claimed

**Published:** 1917-04

**Authors:** 


### Cancer Cure Claimed.

If the experiments of the Rockefeller Institute scientists on cancer cure are confirmed by time, it will be a splendid triumph for modern medicine. These experiments tend to show that the leucocytes, or so-called white blood cells, have the power, when enormously increased in number, of making one immune to cancer. And the Rockefeller Institute men have found a way to double the average number of such cells in the system. Of course, this may be followed by some consequent ill. For one can hardly believe that a doubling of these white lymph cells can be brought about without some after effect. The white lymph cells have an apparent individuality of their own. They wander through the arteries and veins, and even through the tissues, seeking their prey, which is all those germs that may be hurtful to the body. They are the guardians of the blood and cellular tissue. If the new method cures cancer, it will have proved that this disease is actually caused by a germ—something denied by many scientists, because the microscope has so far failed to locate the germ.

